# Genetic interrelationships of North American populations of giant liver fluke *Fascioloides magna*

**DOI:** 10.1186/s13071-015-0895-1

**Published:** 2015-05-28

**Authors:** Eva Bazsalovicsová, Ivica Králová-Hromadová, Jan Štefka, Gabriel Minárik, Silvia Bokorová, Margo Pybus

**Affiliations:** Institute of Parasitology, Slovak Academy of Sciences, Hlinkova 3, 04001 Košice, Slovakia; Biology Centre AS CR, Institute of Parasitology and Faculty of Science, University of South Bohemia, Branišovská 31, 37005 České Budějovice, Czech Republic; Department of Molecular Biology, Faculty of Natural Sciences, Comenius University, Mlynská dolina B-2, 84215 Bratislava, Slovakia; Geneton Ltd., Ilkovičova 3, 84104 Bratislava, Slovakia; Alberta Fish and Wildlife Division and Department of Biological Sciences, University of Alberta, Edmonton, AB Canada

**Keywords:** Trematoda, Fasciolidae, Mitochondrial DNA, Cytochrome *c* oxidase, Nicotinamide dehydrogenase, Spatial distribution

## Abstract

**Background:**

Population structure and genetic interrelationships of giant liver fluke *Fascioloides magna* from all enzootic North American regions were revealed in close relation with geographical distribution of its obligate definitive cervid hosts for the first time.

**Methods:**

Variable fragments of the mitochondrial cytochrome *c* oxidase subunit I (*cox*1; 384 bp) and nicotinamide dehydrogenase subunit I (*nad*1; 405 bp) were applied as a tool. The concatenated data set of both *cox*1 and *nad*1 sequences (789 bp) contained 222 sequences that resulted in 50 haplotypes. Genetic data were analysed using Bayesian Inference (BI), Maximum Likelihood (ML) and Analysis of Molecular Variance (AMOVA).

**Results:**

Phylogenetic analysis revealed two major clades of *F. magna*, which separated the parasite into western and eastern populations. Western populations included samples from Rocky Mountain trench (Alberta) and northern Pacific coast (British Columbia and Oregon), whereas, the eastern populations were represented by individuals from the Great Lakes region (Minnesota), Gulf coast, lower Mississippi, and southern Atlantic seaboard region (Mississippi, Louisiana, South Carolina, Georgia, Florida) and northern Quebec and Labrador. Haplotype network and results of AMOVA analysis confirmed explicit genetic separation of western and eastern populations of the parasite that suggests long term historical isolation of *F. magna* populations*.*

**Conclusion:**

The genetic makeup of the parasite’s populations correlates with data on historical distribution of its hosts. Based on the mitochondrial data there are no signs of host specificity of *F. magna* adults towards any definitive host species; the detected haplotypes of giant liver fluke are shared amongst several host species in adjacent populations.

## Background

Spatial distribution of parasites is closely coupled with distribution of their hosts; parasites require suitable hosts for nutrients and other resources, and thus they cannot occur in territories where susceptible hosts are absent. Therefore, the maximum geographical range of a parasite must equal the combined ranges of its hosts. The hosts also represent the only mean of dispersal for the majority of parasitic taxa and in the case of parasites with multi-host lifecycles, the parasite’s dispersal is driven by its most mobile host. Furthermore, geographical distribution and relationships of the parasites of terrestrial hosts may be affected by historical contractions and expansions of their hosts’ distribution, including recent bottlenecks caused by humans [1, 2]. On the larger scale, a positive relationship between host and parasite species richness is inevitable and generally supported, since most host species harbour at least one host specific parasite [3].

Influence of hosts and parasites on their respective biodiversity is of particular interest, since each partner in a host-parasite association potentially exerts a selective pressure on the other [4]. The relationship between host and parasite represents an intimate interaction between at least two genetic systems [5]. The host-parasite interaction is based on subtle interplay between parasite survival strategies and host defence mechanisms [5]. Revealing population genetic structure and host specificity of the parasitic species in question is the first natural step towards understanding the underlying processes of natural selection.

Giant liver fluke, *Fascioloides magna*, represents a very interesting parasitic model characterized by a wide spectrum of intermediate and definitive hosts, large spatial distribution, potential to colonize new territories and adapt to new host species. It parasitizes a wide range of definitive ruminant hosts, especially cervids. The fluke has a strong predilection to liver parenchyma where it is localized in thin-walled fibrous pseudocysts. It is generally accepted that *F. magna* is of North American origin and may have co-evolved with ancestral cervids, *Odocoileus* spp. [6]. Currently, *F. magna* is enzootic in five major areas across the United States and Canada: (1) the northern Pacific coast, (2) the Rocky Mountain trench, (3) northern Quebec and Labrador, (4) the Great Lakes region, and (5) Gulf coast, lower Mississippi, and southern Atlantic seaboard.

Concerning the host spectrum of giant liver fluke in North America, a variety of free-living (e.g., wapiti *Cervus elaphus canadensis*, white-tailed deer *Odocoileus virginianus*, caribou *Rangifer tarandus*, black-tailed deer *Odocoileus hemionus columbianus* and mule deer *Odocoileus hemionus hemionus*) and domestic ruminants (cattle *Bos taurus*, goat *Capra hircus*, and sheep *Ovis aries*) are known to be susceptible to *F. magna* infection but only white-tailed deer, caribou, and wapiti contribute significantly to maintaining its population [6]. It is assumed that the parasite was widespread in white-tailed deer in major wetland habitats throughout North America; however, the interrelationships of this host with *F. magna* were finely tuned due to low number of flukes within individual deer. On the other hand, potential for translocation of liver flukes in wapiti is higher due to increased *F. magna* egg production and subsequent release to the environment [6].

In Europe in the 19^th^ and 20^th^ centuries, game keeping and hunting had a tendency to increase the biodiversity of game species in local hunting grounds by extensive introduction and restocking of “exotic” game. As a consequence of the introduction of wapiti from North America, the giant liver fluke was unintentionally introduced to Europe, where it established three natural foci of infection; (1) northern Italy, (2) Czech Republic and south-western Poland, and (3) Danube floodplain forests (for review see [7]). In Europe, *F. magna* is considered to be an introduced invasive species with high potential to colonize new geographic territories and establish local populations. In particular, the Danube floodplain forests represent an expanding natural focus of fascioloidosis with inevitable spread of the parasite down the Danube River [8]. In Europe, giant liver fluke has shown high capability to adapt to different intermediate aquatic snail hosts, such as species of *Galba*, *Lymnaea,* and *Radix* [9–11] and definitive ruminant species, in particular red deer *Cervus elaphus elaphus*, roe deer *Capreolus capreolus*, and fallow deer *Dama dama* [10, 12].

The origin of European populations of *F. magna* and the subsequent course of colonisation and migratory routes of this alien parasite in Europe recently were unravelled [13]. Phylogenetic analyses based on concatenated *cox*1 + *nad*1 datasets revealed two genetically separated clades of European *F. magna*. The Italian population represented one phylogenetic clade while the second one included populations from the Czech focus and the Danube floodplain forests. Results clearly indicated that *F. magna* was introduced from North America to Europe at least twice; first to Italy and independently to the Czech Republic. As comparative samples, several *F. magna* specimens from North America were included in the above-mentioned work. The overall analysis of North American and European giant liver fluke populations clearly confirmed a western North American origin of the Italian population; these flukes clustered with specimens from Alberta (Canada) and Oregon (USA). On the other hand, representatives of the Czech Republic and Danube floodplain forests displayed close genetic relationships with parasites from the south-eastern USA. These data showed for the first time relatively high genetic molecular diversity of North American *F. magna* individuals [13]. However, the limited number of the flukes from North America did not cover all five enzootic regions and were insufficient for detailed phylogeographic analysis. Therefore, the aim of the current work was to determine population structure of *F. magna* from a more extensive dataset including all five enzootic North American regions using previously applied mitochondrial *cox*1 and *nad*1 molecular markers [13, 14] in order to reveal genetic interrelationships of giant liver fluke on its original continent. Results were assessed in relation to the geographical distribution of its obligate definitive cervid hosts.

## Methods

### Parasite samples

A total of 248 *F. magna* from 37 livers originating from all five North American enzootic regions were included in the analysis (Table 1). From the 248 parasites, 140 samples were newly analysed, while the remaining ones were already included in our previous analysis [13] (see Table 1, superscript a). Flukes were isolated directly from parenchymatous cysts and immediately rinsed in PBS buffer and fixed in 96 % ethanol.

**Table 1:** Details on *Fascioloides magna* specimens analyzed in the current study

Enzootic region/Region code	Country/province (state)	Locality	Geographical coordinates	Host	Liver code	Number of flukes
Northern Quebec and Labrador/NQL	Canada/Quebec (QC)	Kuujjuaq	58°44'N, 70°02'W	*Ovibos moschatus*	QC-1	6
		Tasiujaq	58°06'N, 68°23'W	(muskox)	QC-2	7
					QC-3	4
					QC-4	2
				***subtotal***	***4***	***19***
Northern Quebec and Labrador/NQL	Canada/ Newfoundland and Labrador (NL)	northern Labrador at Nashaupi River	53°55'N, 60°44'W	*Rangifer tarandus*	NL-1	2
				(caribou)	NL-2	2
					NL-3	2
				***subtotal***	***3***	***6***
Rocky Mountains trench/RMT	Canada/Alberta (AB)	Banff National Park	51°12'N, 115°35'W	*Cervus elaphus canadensis*	AB-2	23
					AB-5	1
				(wapiti; Rocky Mountain elk)	AB-8	17
					AB-11	9
					AB-1	37^a^
					AB-4	1^a^
					AB-6	1^a^
					AB-8	5^a^
					AB-9	8^a^
					AB-11	5^a^
					AB-15	1^a^
				***subtotal***	***9***	***108***
Northern Pacific Coast/NPC	Canada/British Columbia (BC)	Vancouver Island	49°00'N, 127°00'W	*Cervus elaphus roosvelti*	BC-1	8
				(wapiti; Roosevelt elk)	BC-2	1
					BC-3	1
					BC-4	2
					BC-5	3
				***subtotal***	***5***	***15***
Northern Pacific Coast/NPC	USA/Oregon (OR)	Salem	44°56'N, 123°02'W	*Odocoileus hemionus columbianus*	OR-33	1
				(black-tailed deer)	OR-33	4^a^
				***subtotal***	***1***	***5***
Great Lakes Region/GLR	USA/Minnesota (MN)	Erskine	47°40'N, 96°00'W	*Odocoileus virginianus*	MN	28^a^
		Hibbing		(white-tailed deer)		
				***subtotal***	***1***or ***>1*** ^b^	***28***
Gulf coast, Lower Mississippi and Southern Atlantic Seaboard/SAS	USA/Mississippi (MS)	St. Catherine NWR	32°32'N, 91°22'W	*C. e. canadensis*	MS-35	2
				(wapiti; Rocky Mountain elk)	MS-35	3^a^
					MS-34	2^a^
				***subtotal***	***2***	***7***
Gulf coast, Lower Mississippi and Southern Atlantic Seaboard/SAS	USA/Louisiana (LA)	Tensas NWR	32°03'N, 91°15'W	*C. e. canadensis*	LA-38	4
				(wapiti; Rocky Mountain elk)	LA-39	2
					LA-38	1^a^
					LA-39	1^a^
				***subtotal***	***2***	***8***
Gulf coast, Lower Mississippi and Southern Atlantic Seaboard/SAS	USA/South Carolina (SC)	Savannah River Site	34°26′N, 82°51′W	*O. virginianus*	SC-40	5
				(white-tailed deer)	SC-41	5
					SC-42	8
					SC-43	7
					SC-44	2
					SC-45	2
				***subtotal***	***6***	***29***
Gulf coast, Lower Mississippi and Southern Atlantic Seaboard/SAS	USA/Georgia (GA)	Wilkinson	32°48'N, 83°09'W	*C. e. canadensis*	GA-31	4
				(wapiti; Rocky Mountain elk)	GA-32	1^a^
				***subtotal***	***2***	***5***
Gulf coast, Lower Mississippi and Southern Atlantic Seaboard/SAS	USA/Florida (FL)	White Oak plantation	30°44'N, 81°45'W	*C. e. canadensis*	FL-36	2
				(wapiti; Rocky Mountain elk)	FL-37	6
					FL-36	2^a^
					FL-37	8^a^
				***subtotal***	***2***	***18***
				**TOTAL**	**37 or > 37** ^b^	**248**

^a^Sequences published by Králová-Hromadová et al. [13]; ^b^ *F. magna* specimens from Minnesota were not identified by liver sample or exact sampling site; NWR, National Wildlife Refuge

Flukes from the enzootic region of northern Quebec and Labrador (NQL) were collected from muskox (*Ovibos moschatus*) and caribou (*Rangifer tarandus*), while specimens from the Rocky Mountain trench (RMT) originated from wapiti. In the northern Pacific coast (NPC), *F. magna* samples were isolated from Roosevelt elk (*Cervus elaphus roosevelti)* and black-tailed deer. In Minnesota, belonging to the Great Lakes region (GLR), all flukes came from white-tailed deer. Finally, the Gulf coast, lower Mississippi, and southern Atlantic seaboard (SAS) enzootic region was represented by *F. magna* from wapiti and white-tailed deer; in particular flukes were obtained from US states of Mississippi (MS), Louisiana (LA), Georgia (GA), Florida (FL), and South Carolina (SC).

### DNA isolation, PCR amplification and sequencing

Genomic DNA was isolated from 20 mg of adult flukes using phenol:chlorophorm:isoamyl alcohol extraction and ethanol precipitation [14]. In order to completely remove remaining PCR inhibitors, such as divalent cations and proteins, two additional wash steps using the QIAamp® DNA Kit (QIAGEN, Hilden, Germany) were employed in the DNA purification procedure. Finally, genomic DNA was diluted in deionised water and stored at −20 °C.

For amplification and sequencing of partial mitochondrial cytochrome *c* oxidase subunit I (*cox*1; 384 bp) and nicotinamide dehydrogenase subunit I (*nad*1; 405 bp), the previously designed primers and PCR conditions were applied [13, 14]. The PCR products were loaded on the 1 % agarose gel and purified either using the Wizard PCR purification Kit (Promega, Madison, Wisconsin) or with exonuclease I and shrimp alkaline phosphatase [15]. Sequencing was performed using an automatic genetic analyzer Applied Biosystems 3130xl (Applied Biosystems, Foster City, California) and BigDye Terminator v3.1 Cycle sequencing kit (Applied Biosystems). Contiguous sequences were assembled and inspected for errors using Geneious version 7.1.7 (Biomatters, Auckland, New Zealand). The translation into the amino-acid sequence was performed according to the trematode mitochondrial code [16, 17]. Concatenation of *cox*1 and *nad*1 datasets was performed in SeaView 4.2 [18]. Distribution maps showing sampling locations were prepared using the Inkscape version 0.485.0 (free download from https://inkscape.org).

### Computational, statistical and phylogenetic analyses of genetic data

Phylogenetic reconstruction of the relationships between mtDNA haplotypes was performed using concatenated data with Maximum Likelihood algorithms (ML) in PhyML 3.0 [19] and Bayesian Inference (BI) in MrBayes 3.2.3 [20]. We used a concatenated dataset based on the experience from a previous analysis [13], where individual gene networks did not provide conflicting patterns. Statistical support of the resulting topologies was obtained by 1000 bootstrap replications in PhyML. MrBayes was run in two parallel runs with four chains each and two million MCMC replications sampled every 1000 generations. Twenty percent of the run was discarded as burn-in. Convergence of the parameters obtained in the two runs was inspected in Tracer 1.6 (http://tree.bio.ed.ac.uk/software/tracer). Model of molecular evolution for the BI analysis was selected in PartitionFinder [21] allowing separate parameter estimation for each gene and each codon position. HKY + I model was selected for the first two codon positions, whereas GTR + G was selected for the third position. TN93 model was selected in PartitionFinder for the PhyML analysis, where the usage of separate models for codon positions is not supported. Phylogenetic trees were rooted using *cox*1 and *nad*1 sequences of the closest available relative, *Fasciola hepatica* (GenBank Accession No. NC002546). The topology used to position a root for the trees from the ML and BI analyses was obtained in PhyML by a ML analysis of translated amino acid sequences under the LG model. Amino acid sequence analysis was used due to a relatively deep level of genetic variation between the two genera, which created a very long branch when analysed in the DNA mode.

Genealogical information contained in the two mtDNA genes was visualized using haplotype network in the software TCS 1.21 [22]. To explore the effect of geographical distribution on the structuring of the populations, analysis of molecular variance (AMOVA) was performed using the concatenated dataset in Arlequin 3.5.1.2 [23]. The hierarchical grouping of samples for the analysis was designed in two alternative schemes: 1) populations were grouped into five enzootic foci [6] of *F. magna* (see Table 1); 2) population groups were reorganized into six sets to better reflect the distribution and sharing of haplotypes between populations seen in TCS network. Regions in the north-west (NPC and RMT) were pooled, whereas the SAS region in the south-east was divided into three separate groups (Table 2). Significance of the obtained results was tested with 10,000 permutations of the data. Analyses of haplotype diversity (*Hd*), nucleotide diversity (*Pi*) and neutrality tests (Tajima’s D, Fu and Li’s D, Fu and Li’s F) for population sets used in the second AMOVA analysis were calculated for concatenated data in DNASP 5.10.1 [24]. Significance of the neutrality tests was obtained with 10,000 coalescent simulations.

**Table 2:** Analysis of molecular variance (AMOVA) of population structure of North American *Fascioloides magna*

	**Fst** (*cox*1 + *nad*1)		
		Variance	
Grouping criterion	d.f.	components	Percent
***Five enzootic regions***			
Among groups	4	0.968	18.25^a^
Among populations within groups	6	2.946	**55.55**
Within populations	209	1.390	**26.20**
***Structured populations***			
(AB, BC, OR) (MS, LA) (FL, GA)			
(SC) (MN) (QC, NL)			
Among groups	5	4.149	**72.99**
Among populations within groups	5	0.145	*2.56*
Within populations	209	1.390	**24.45**

Codes of US states, Canadian provinces and enzootic regions are explained in Table 1; AB, BC, OR - RMT and NPC enzootic regions; MS, LA – 1^st^ group of SAS region; FL, GA – 2^nd^ group of SAS; SC – 3^rd^ group of SAS; MN – GLR region; QC, NL – NQL region; Fst, F-statistics; d.f., degrees of freedom; ^a^insignificant results (P > 0.05); results significant at P < 0.001 are in bold; results significant at P < 0.05 are in italics

## Results

The analysis of 384 bp *cox*1 mtDNA fragment (128 amino acids, aa) and 405 bp *nad*1 fragment (134 aa + stop codon) revealed 32 *cox*1 (CO1-Ha) and 28 *nad*1 (ND1-Ha) mitochondrial haplotypes (Table 3). The numbering of CO1-Ha and ND1-Ha haplotypes, as presented in Table 3, adopted the strategy of numbering applied in our recently published study on genetic interrelationship of European populations of *F. magna* [13]. The newly determined haplotypes of North American *F. magna* respected the numbering of the mentioned work and continuously proceeded in numbering new haplotypes. Since three *cox*1 (CO1-Ha2, 4, and 5) and four *nad*1 (ND1-Ha1, 2, 5, and 7) haplotypes were detected exclusively in the European populations of the parasite [13] they are not presented in Table 3.

**Table 3:** The *cox*1 (CO1-Ha) and *nad*1 (ND1-Ha) haplotypes identified for *Fascioloides magna* from North American localities

Country/province, state	*cox*1 haplotype code	GenBank Acc. no.	No. of specimens	*nad*1 haplotype code	GenBank Acc. no.	No. of specimens
			CO1-Ha freq. - %			ND1-Ha freq. - %
Canada/Alberta (AB)	CO1-Ha1/AB	GU599861^a^	95 → 38.3 (Ha1)	ND1-Ha3/AB	GU599845^a^	90 → 39.6 (Ha3)
	CO1-Ha6/AB	GU599871^a^	26 → 10.5 (Ha6)	ND1-Ha8/AB	GU599846^a^	11 → 4.8 (Ha8)
Canada/British	CO1-Ha1/BC	KP635011		ND1-Ha3/BC	KP635037	
Columbia (BC)	CO1-Ha20/BC	KP635012	3 → 1.2 (Ha20)	ND1-Ha24/BC	KP635038	6 → 2.6 (Ha24)
	CO1-Ha21/BC	KP635013	5 → 2.0 (Ha21)	ND1-Ha25/BC	KP635039	3 → 1.3 (Ha25)
				ND1-Ha30/BC	KP635040	1 → 0.4 (Ha30)
Canada/Northern	CO1-Ha9/NL/QC	KP635014, KP635017	14 → 5.6 (Ha9)	ND1-Ha12/QC	KP635041	11 → 4.8 (Ha12)
Quebec (QC) and	CO1-Ha17NL/QC	KP635015, KP635018	10 → 4.0 (Ha17)	ND1-Ha19/QC/NL	KP635042, KP635047	25 → 11.0 (Ha19)
Labrador (NL)	CO1-Ha18/NL	KP635016	1 → 0.4 (Ha18)	ND1-Ha20/QC/NL	KP635043, KP635048	6 → 2.6 (Ha20)
	CO1-Ha22/QC	KP635019	5 → 2.0 (Ha22)	ND1-Ha21/QC	KP635044	2 → 0.9 (Ha21)
	CO1-Ha28/QC	KP635020	2 → 0.8 (Ha28)	ND1-Ha22/QC/NL	KP635045, KP635049	4 → 1.8 (Ha22)
				ND1-Ha23/QC	KP635046	1 → 0.4 (Ha23)
USA/Oregon (OR)	CO1-Ha1/OR	GU599862^a^		ND1-Ha3/OR	GU599848^a^	
USA/Minnesota (MN)	CO1-Ha8/MN	GU599873^a^	13 → 5.2 (Ha8)	ND1-Ha9/MN	GU599849^a^	7 → 3.1 (Ha9)
	CO1-Ha9/MN	GU599874^a^		ND1-Ha10/MN	GU599850^a^	12 → 5.3 (Ha10)
	CO1-Ha10/MN	GU599875^a^	4 → 1.6 (Ha10)	ND1-Ha11/MN	GU599851^a^	2 → 0.9 (Ha11)
	CO1-Ha11/MN	GU599876^a^	3 → 1.2 (Ha11)	ND1-Ha12/MN	GU599852^a^	
	CO1-Ha19/MN	KP635021	1 → 0.4 (Ha19)	ND1-Ha32/MN	KP635050	1 → 0.4 (Ha32)
USA/Mississippi (MS)	CO1-Ha9/MS	KP635022		ND1-Ha12/MS	KP635051	
	CO1-Ha12/MS	GU599877^a^	2 → 0.8 (Ha12)	ND1-Ha15/MS	GU599855^a^	2 → 0.9 (Ha15)
	CO1-Ha13/MS	GU599878^a^	1 → 0.4 (Ha13)	ND1-Ha16/MS	GU599856^a^	2 → 0.9 (Ha16)
	CO1-Ha14/MS	GU599879^a^	1 → 0.4 (Ha14)	ND1-Ha17/MS	GU599857^a^	5 → 2.2 (Ha17)
	CO1-Ha15/MS	GU599880^a^	3 → 1.2 (Ha15)			
USA/Florida (FL)	CO1-Ha16/FL	GU599882^a^	22 → 8.9 (Ha16)	ND1-Ha13/FL	GU599853^a^	15 → 6.6 (Ha13)
				ND1-Ha14/FL	GU599854^a^	2 → 0.9 (Ha14)
				ND1-Ha27/FL	KP635052	3 → 1.3 (Ha27)
				ND1-Ha28/FL	KP635053	2 → 0.9 (Ha28)
USA/Georgia (GA)	CO1-Ha7/GA	GU599872^a^	1 → 0.4 (Ha7)	ND1-Ha9/GA	GU599847^a^	
	CO1-Ha16/GA	KP635023		ND1-Ha13/GA	KP635054	
USA/Louisiana (LA)	CO1-Ha15/LA	GU599881^a^		ND1-Ha10/LA	KP635055	
	CO1-Ha29/LA	KP635024	1 → 0.4 (Ha29)	ND1-Ha12/LA	KP635056	
	CO1-Ha30/LA	KP635025	1 → 0.4 (Ha30)	ND1-Ha16/LA	GU599858^a^	
	CO1-Ha31/LA	KP635026	1 → 0.4 (Ha31)	ND1-Ha17/LA	KP635057	
	CO1-Ha32/LA	KP635027	1 → 0.4 (Ha32)	ND1-Ha18/LA	GU599859^a^	2 → 0.9 (Ha18)
	CO1-Ha33/LA	KP635028	2 → 0.8 (Ha33)			
	CO1-Ha34/LA	KP635029	1 → 0.4 (Ha34)			
USA/South Carolina (SC)	CO1-Ha3/SC	KP635030	7 → 2.8 (Ha3)	ND1-Ha4/SC	KP635058	2 → 0.9 (Ha4)
	CO1-Ha23/SC	KP635031	16 → 6.5 (Ha23)	ND1-Ha6/SC	KP635059	5 → 2.2 (Ha6)
	CO1-Ha24/SC	KP635032	1 → 0.4 (Ha24)	ND1-Ha19/SC	KP635060	
	CO1-Ha25/SC	KP635033	1 → 0.4 (Ha25)	ND1-Ha26/SC	KP635061	1 → 0.4 (Ha26)
	CO1-Ha26/SC	KP635034	1 → 0.4 (Ha26)	ND1-Ha29/SC	KP635062	3 → 1.3 (Ha29)
	CO1-Ha27/SC	KP635035	2 → 0.8 (Ha27)	ND1-Ha31/SC	KP635063	1 → 0.4 (Ha31)
	CO1-Ha35/SC	KP635036	1 → 0.4 (Ha35)			

Frequency (freq.) of haplotypes were calculated for all individuals having the respective Ha despite of their locality; numbering of haplotypes follows that of Králová-Hromadová et al. [13], therefore Ha numbers of exclusively European populations are missing; ^a^data published in Králová-Hromadová et al. [13]

**Fig. 1:**
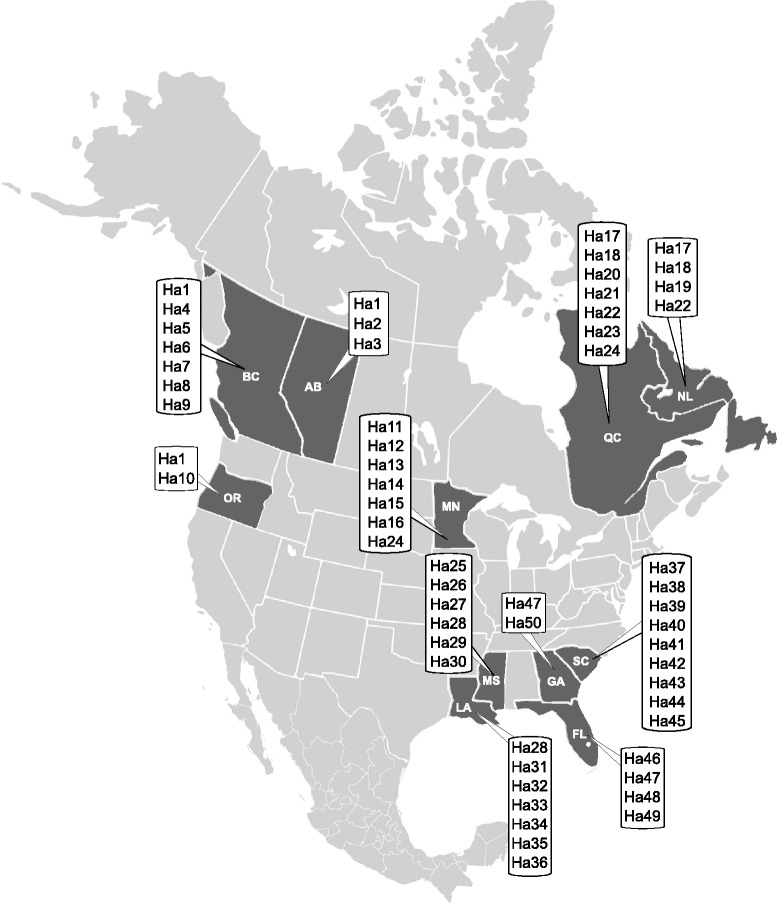
Distribution of concatenated mtDNA haplotypes (for details see Table 4) of *Fascioloides magna* in North America. Geographic origin of *F. magna* individuals analysed in the current work is displayed as dark grey regions. Codes for US states and Canadian provinces are explained in Table 1

Of 43 polymorphic sites detected in *cox*1 (transitions (ts)/transversions (tv) ratio; 40/3), 36 substitutions were not responsible for change in amino acid sequence while seven mutations underwent the non-synonymous substitutions. As for *nad*1, 16 substitutions out of 23 (ts/tv ratio; 19/4) were silent whereas seven mutations changed the amino acid sequence. The concatenated dataset of both *cox*1 and *nad*1 sequences (789 bp) contained 222 sequences that resulted in 50 haplotypes (Table 4; Fig. 1). The concatenated dataset contained 63 variable characters, of which 42 characters were parsimony-informative.

**Table 4:** Details on concatenated haplotypes (*cox*1 + *nad*1) of North American *Fascioloides magna* individuals

Concatenated haplotype	US state, CA province	*cox*1 haplotype	*nad*1 haplotype
Ha1	AB, BC, OR	CO1-Ha1	ND1-Ha3
Ha2	AB	CO1-Ha1	ND1-Ha8
Ha3	AB	CO1-Ha6	ND1-Ha3
Ha4	BC	CO1-Ha21	ND1-Ha3
Ha5	BC	CO1-Ha20	ND1-Ha30
Ha6	BC	CO1-Ha1	ND1-Ha24
Ha7	BC	CO1-Ha21	ND1-Ha24
Ha8	BC	CO1-Ha20	ND1-Ha25
Ha9	BC	CO1-Ha1	ND1-Ha25
Ha10	OR	CO1-Ha1	ND1-Ha3
Ha11	MN	CO1-Ha19	ND1-Ha9
Ha12	MN	CO1-Ha8	ND1-Ha32
Ha13	MN	CO1-Ha11	ND1-Ha9
Ha14	MN	CO1-Ha10	ND1-Ha9
Ha15	MN	CO1-Ha10	ND1-Ha11
Ha16	MN	CO1-Ha8	ND1-Ha10
Ha17	NL, QC	CO1-Ha17	ND1-Ha19
Ha18	NL, QC	CO1-Ha9	ND1-Ha20
Ha19	NL	CO1-Ha18	ND1-Ha19
Ha20	QC	CO1-Ha22	ND1-Ha23
Ha21	QC	CO1-Ha28	ND1-Ha12
Ha22	NL, QC	CO1-Ha22	ND1-Ha22
Ha23	QC	CO1-Ha17	ND1-Ha21
Ha24	MN, QC	CO1-Ha9	ND1-Ha12
Ha25	MS	CO1-Ha12	ND1-Ha15
Ha26	MS	CO1-Ha13	ND1-Ha17
Ha27	MS	CO1-Ha14	ND1-Ha17
Ha28	MS, LA	CO1-Ha15	ND1-Ha16
Ha29	MS	CO1-Ha9	ND1-Ha17
Ha30	MS	CO1-Ha15	ND1-Ha12
Ha31	LA	CO1-Ha32	ND1-Ha12
Ha32	LA	CO1-Ha29	ND1-Ha17
Ha33	LA	CO1-Ha30	ND1-Ha12
Ha34	LA	CO1-Ha34	ND1-Ha10
Ha35	LA	CO1-Ha33	ND1-Ha18
Ha36	LA	CO1-Ha31	ND1-Ha17
Ha37	SC	CO1-Ha24	ND1-Ha31
Ha38	SC	CO1-Ha25	ND1-Ha19
Ha39	SC	CO1-Ha23	ND1-Ha19
Ha40	SC	CO1-Ha3	ND1-Ha6
Ha41	SC	CO1-Ha26	ND1-Ha6
Ha42	SC	CO1-Ha35	ND1-Ha29
Ha43	SC	CO1-Ha27	ND1-Ha29
Ha44	SC	CO1-Ha3	ND1-Ha4
Ha45	SC	CO1-Ha23	ND1-Ha26
Ha46	FL	CO1-Ha16	ND1-Ha14
Ha47	FL, GA	CO1-Ha16	ND1-Ha13
Ha48	FL	CO1-Ha16	ND1-Ha27
Ha49	FL	CO1-Ha16	ND1-Ha28
Ha50	GA	CO1-Ha7	ND1-Ha9

Codes of US states and Canadian (CA) provinces are explained in Table 1; details on individual CO1-Ha and ND1-Ha are presented in Table 3

**Fig. 2:**
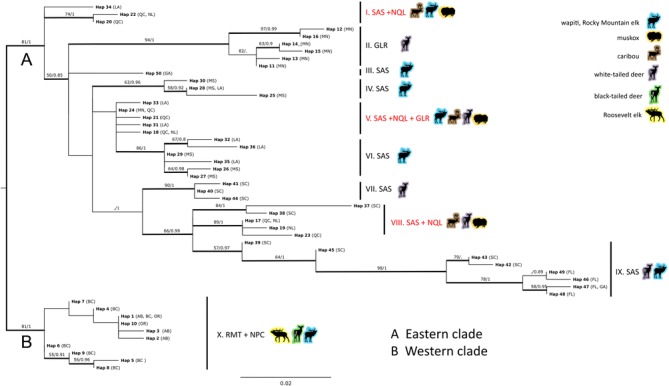
Maximum likelihood phylogeny of *Fascioloides magna* concatenated mitochondrial haplotypes. Bootstrap/posterior probability statistics higher than 50 % and 0.80, respectively, are provided above respective branches in bold. Codes for enzootic regions, US states and Canadian provinces are explained in Table 1. Phylogeny was rooted using a separate amino acid sequence analysis in PhyML using *Fasciola hepatica* as outgroup (outgroup not shown)

Phylogenetic analysis revealed two major clades (Fig. 2), one comprising samples from western enzootic regions, Rocky Mountain trench (RMT) and northern Pacific coast (NPC) (Canadian provinces BC, AB, and US state OR) (Fig. 2, clade B) whereas the second clade comprised samples from eastern enzootic regions, specifically the Great Lakes region (GLR) (US state MN), Gulf coast, lower Mississippi, and southern Atlantic seaboard enzootic region (SAS) (US states MS, LA, SC, GA, FL) and northern Quebec and Labrador (NQL) (Canadian provinces QC and NL) (Fig. 2, clade A).

The spectrum of definitive hosts sampled in the western enzootic regions was limited to three cervid hosts: wapiti, Roosevelt elk, and black-tailed deer. The respective samples of the western clade (US state OR, Canadian provinces AB and BC) were characterized by single cervid hosts; AB/wapiti, BC/Roosevelt elk, and OR/black-tailed deer (Table 1). However, as evident from the internal structure of the phylogenetic clade B, the interrelationships of western *F. magna* population were not related to cervid host species.

The second dominant phylogenetic clade (clade A) of eastern enzootic regions was polyphyletic. Some of the respective sublineages within clade A mirror the geography/host pattern. The most homogenous internal lineages were no. 2 (GLR-Minnesota; white-tailed deer), nos. 4 and 6 (SAS-Louisiana, Mississippi; wapiti), and no. 7 (SAS-South Carolina; white-tailed deer) (Fig. 2). One specimen from Georgia (SAS) (Ha50, lineage 3), clustered separately. Lineage no. 9 was created by haplotypes specific to SAS (US states SC, FL, GA) and two hosts; wapiti and white-tailed deer. On the other hand, lineage nos. 1 and 8 were heterologous; haplotypes within these lineages corresponded to *F. magna* from geographically distant regions – SAS and NQL. Even more diverse was lineage no. 5, which included representatives of all eastern populations: SAS, NQL and GLR. Additionally, besides heterogeneity in geography, the three heterologous lineages (1, 5 and 8) were characterized by the most diverse spectrum of definitive hosts – wapiti, caribou, muskox and white-tailed deer (Fig. 2).

**Fig. 3:**
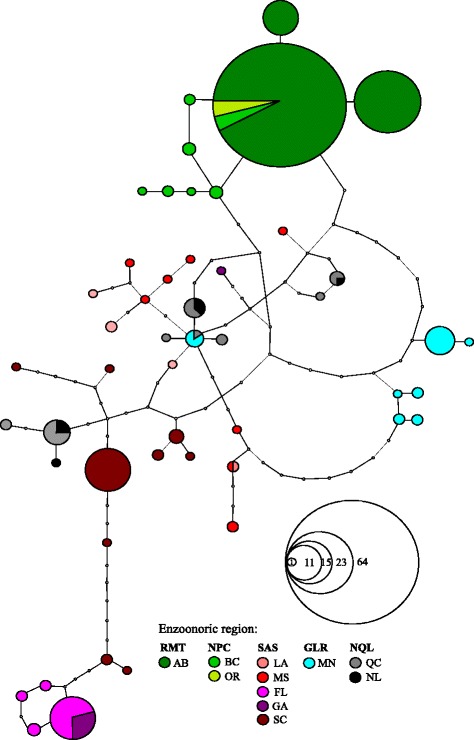
Haplotype network of *Fascioloides magna* populations obtained in TCS. Each haplotype is represented by a circle scaled according to the number of specimens. Empty circles along the mutation pathways represent putative unsampled haplotypes. Codes for enzootic regions, US states and Canadian provinces are explained in Table 1

The mitochondrial network also revealed frequent sharing of haplotypes between western enzootic regions NPC and RMT, and relative isolation of populations from Minnesota and Florida (Fig. 3). Although haplotypes specific for SAS and NQL did not create separate clusters in the network, haplotypes of these two eastern enzootic regions were not shared among different geographic areas. The only exception was Ha24, which was shared between MN (GLR) and QC (NQL) (Table 4). Regardless, samples from different locations within SAS had very little overlap. Haplotypes were shared within but not among three groups (SC + FL, GA, MS + LA) with one exception of shared haplotype between Florida and Georgia. On the contrary, populations in the north-eastern enzootic focus (NQL) shared most of their haplotypes between Quebec and Labrador despite different host origin (caribou and muskox). Surprising overlap in haplotypes was detected for individuals from Minnesota (GLR) and Quebec (NQL). Other haplotypes did not cluster according to host origin either. For example haplotypes were shared among wapiti and black-tailed deer in Alberta and Oregon.

The AMOVA analysis reflects the pattern obtained from phylogenetic analysis and haplotype network. Populations grouped according to their enzootic membership explained very little variance in the data, whereas variance in data within the areas was much higher (Table 2). On the contrary, populations organized into six groups according to the distribution of the haplotypes explained almost 73 % of the variance at the highest level (among areas), whereas less than 3 % was left among populations within the areas. Characterization of the same six groups performed in DNASP revealed some differences. Most of the population groups had similar levels of *Hd* and *Pi* diversities and non-significant results of neutrality tests (Table 5). Only the western population (AB, BC, OR) and the SAS2 group (FL, GA) had lower values of *Hd* and *Pi*. The SAS2 group also had significantly negative values in all three tests of neutrality. Negative values of neutrality tests usually are interpreted as an indication of population expansion after a bottleneck or a selective sweep (e.g., [25]).

**Table 5:** Molecular variability and neutrality tests for six defined North American areas of *Fascioloides magna*

Area	S. size	No. Ha	Hd	Pi	Tajima's D	Fu and Li's D	Fu and Li's F
**NPC+RMT** (AB, BC, OR)	110	9	0.61	0.0011	-0.504	1.112	0.676
**SAS1** (LA, MS)	15	11	0.96	0.0074	-0.584	-0.505	-0.606
**SAS2** (FL, GA)	23	5	0.57	0.0029	-1.733^a^	**-2.883**	**-2.960**
**SAS3** (SC)	28	9	0.70	0.0065	-0.317	0.242	0.077
**GLR** (MN)	21	7	0.79	0.0066	0.637	1.208	1.209
**NQL** (QC, NL)	25	8	0.83	0.0078	1.256	1.232	1.449

Codes of US states and Canadian provinces are explained in Table 1; S. size, sample size; No. Ha, number of haplotypes; Hd, haplotype diversity; Pi, nucleotide diversity; ^a^results significant at P < 0.05; results significant at P < 0.01 are in bold

## Discussion

Current data provide a complete picture of the genetic structure of *F. magna* populations from all enzootic regions in North America (NA); the Rocky Mountain trench (RMT), the northern Pacific coast (NPC), northern Quebec and Labrador (NQL), the Great Lakes region (GLR), and the Gulf coast, lower Mississippi, and southern Atlantic seaboard (SAS). The most straightforward finding was explicit genetic separation of western (NPC and RMT) and eastern (NQL, GLR and SAS) populations of the parasite.

Parasites in general rely almost entirely on their hosts for dispersal ranging from small to large scales [3]. The geographical distributions of most parasite species are limited by the distributions of potential host species or by environmental constraints on the parasite’s rates of development [26]. The population genetic structure of a parasite, and consequently its ability to adapt to a given host, is strongly linked to its own life history as well as the life history of its host [27]. From this point of view, relationships of genetically and demographically variable hosts and parasites should be studied in direct collaboration [4].

The genetic structure of NA giant liver fluke populations needs to be related to the historical and current distribution of obligate definitive NA cervid hosts of *F. magna*, in particular wapiti, white-tailed deer, and caribou. According to Pybus [6], current NA populations of *F. magna* are separated in detached pockets across North America and the parasite may have co-evolved with the ancestral *Odocoileus* spp. Giant liver fluke has originally been widespread in white-tailed deer in major wetland habitats throughout NA where wapiti and caribou sympatric with white-tailed deer encountered *F. magna* in overlapping contaminated regions.

The distinction of two mitochondrial lineages of *F. magna* corresponds very probably to historical distribution, extirpation, re-introduction, and current occurrence of two obligate definitive hosts of giant liver fluke; white-tailed deer in eastern and wapiti in the western part of NA. *Odocoileus* spp. is native to North America and its populations were well established in the south-eastern USA during the Pleistocene epoch [28]. In the early part of the 19^th^ century unrestricted hunting, deforestation and extensive agricultural development led to dramatic declines in the white-tailed deer populations in the south-eastern USA [29]. Implementation of an extensive restocking programme during the late 19^th^ and in 20^th^ century increased the white-tailed deer populations, especially in the south-east where re-establishing deer populations was considered essential [30].

On the other hand, wapiti is of Eurasian origin and represents phylogenetically the most derived “old world deer”, which displayed different migratory routes to the NA continent with the postglacial reopening of the passage southward from Beringia into the mid-continent [31]. Generally, wapiti were extirpated from the Rocky Mountains of Alberta and British Columbia by the early 1900s and reduced to a few remnant populations in isolated areas. In the first half of the 20^th^ century, wapiti were restocked from the Yellowstone National Park and expanded in number and geographic distribution within the Banff National Park (BNP). A significant exchange of wapiti between BNP and Kootenay National Park through Vermilion Pass provided a natural dispersal mechanism for *F. magna* from British Columbia into Alberta (see [32] and references therein).

Western populations of wapiti and populations of white-tailed deer in the eastern part of NA are separated by an expansive area of dry grasslands (the Great Plains region) throughout the core of Canada and USA, with very limited ecological conditions suitable for maintaining the giant liver fluke. Consequently, western and eastern populations of *F. magna* might be separated on a historical timeframe and evolved distinct genetic structure. Alternatively, the apparent western and eastern *F. magna* population structure may be a result of the lack of suitable cervid hosts following widespread extirpation of ungulate populations in eastern and central NA following European colonization that may also reduce the opportunity for genetic admixing among local *F. magna* populations [6]. The effect on population segregation has been documented by studies of mtDNA phylogeography in white-tailed deer [30, 33] as well as two other hosts of *F. magna*, the black-tailed deer [34] and caribou [35]. However, we find the historical separation more probable. The effect of such a recent bottleneck (<200 years ago) would have to be extremely strong to produce reciprocal monophyly between the two population clades A and B. Furthermore, we would expect to see strongly reduced numbers of haplotypes in local populations of *F. magna*. Neither the diversity of obtained haplotypes (Fig. 3) nor the results of neutrality tests (Table 5) point to such a scenario.

Phylogenetic reconstruction of the relationships between concatenated mtDNA haplotypes using Maximum Likelihood and Bayesian Inference, as well as haplotype network reflecting genealogical information congruently revealed genetic admixture of *F. magna* individuals from geographically distant eastern populations of the parasite (NQL, GLR, SAS), where overlapping distribution of white-tailed deer and caribou may have played an important role. Caribou were present in North America as early as the glacial periods of the middle Pleistocene in Beringia [36]. In the 19^th^ century, peripheral populations of caribou within the United States were extirpated and populations that occurred from Minnesota to Maine, in New York, Wisconsin and Michigan disappeared [37, 38]. While white-tailed deer was widespread in major wetland habitats throughout NA, eastern populations of caribou ranged as far south as Alabama in eastern NA [39] and overlapped with white-tailed deer in the Great Lakes region [40, 41]. Consequently, caribou sympatric with white-tailed deer could have encountered *F. magna* in overlapping contaminated wetland habitats, thus facilitating its movement into new areas and establishing new parasite populations. The present fluke population in caribou in the northeast (NQL) is most probably a residual population that survived the caribou extirpations in more southern regions. In addition, giant liver fluke further spilled over into local muskox populations that were overlapping with infected caribou in NQL region.

In contrast to the general pattern of mixing in the eastern populations, some populations seem to retain genetic distinctiveness. For example, all but one haplotype from Florida and Georgia created a separate cluster in the haplotype network (Fig. 3). Despite the samples originated from wapiti, which was probably introduced to the area recently, white-tailed deer was the naturally occurring host in the past. In the mitochondrial analysis by Ellsworth et al. [30] the populations of *O. virginianus* from south Florida showed genetic differentiation attributable to pleistocene climatic oscillations [30]. Thus, current genetic patterns of the parasite in the same region may be remnants of past population structure the host. In relation to that it is noteworthy that the FL + GA fluke population was the only one where significantly negative values of neutrality tests were seen (Table 5).

Pertinent to the genetic relatedness of western populations of giant liver fluke (NPC and RMT) confirmed by statistical testing (AMOVA analysis) and phylogenetic analyses (Maximum Likelihood and Bayesian Inference), the distribution of six populations (subspecies) of *Cervus elaphus* in North America [41] needs to be considered. The Roosevelt elk population (*C. e. roosevelti*) along the Pacific coast of British Columbia and US states Washington/Oregon (NPC enzootic region) is immediately adjacent to the Rocky Mountain elk (*C. e. canadensis*) population in the RMT enzootic region. The Coastal Mountains to the west (where the Roosevelt elk originated) are separated from the Rocky Mountains to the east (origin of Rocky Mountain elk) only by a broad lowland area (the Interior Plateau) [41], which provides an extensive network of contiguous forests, wetlands, lakes and rivers. This biotope offers shared habitats for different populations of wapiti in western enzootic regions NPC and RMT and also suitable environmental conditions for perpetuation of *F. magna* populations. The current shared genetic makeup of the western populations of *F. magna* could be explained by admixing of the fluke population in association with distribution of different wapiti subspecies in this region.

Based on the mitochondrial data, it can be concluded that there are no signs of host specificity of *F. magna* adults towards any definitive host species; the detected haplotypes of giant liver fluke are shared amongst several host species in adjacent populations. Similar genetic patterns of geographic isolation with shared hosts broadly displayed e.g., winter ticks *Dermacentor albipictus* on cervids in North America [42], suggests widespread patterns of historic factors directly affecting multiple host parasite relationships.

## Conclusion

Genetically diverse NA populations of *F. magna* reflect historical distribution, past extirpation, subsequent re-introduction, and current occurrence of the obligate definitive cervid hosts of giant liver fluke. The present study provides missing pieces of the puzzle and completes the comprehensive dataset on mitochondrial structure and population diversity of all (NA and European) *F. magna* populations. Comparison of original results with published data [13] revealed the following. Since North America is the original continent of *F. magna*, a high level of molecular diversity in mitochondrial haplotypes evident between and within respective enzootic regions was anticipated. While the total number of concatenated haplotypes in NA populations ranged from three (RMT) and eight (each in NQL and NPC) to 26 (SAS) (present study) only two haplotypes were determined in Danube floodplain forests and four in each of Italy and the Czech Republic [13]. As expected, lower genetic heterogeneity was determined in newly established natural foci after introduction of non-indigenous species and the bottleneck effect is evident (data not shown).

The only haplotype common for parasites from both continents was haplotype 1, assessed in *F. magna* from Italy, Alberta, and Oregon, thus confirming the western NA origin of fascioloidosis in the first European focus – Italy [13]. After comparison of present data with data achieved for European *F. magna* samples [13] two additional haplotypes (Ha40, Ha44; Table 4) detected in specimens from South Carolina (SAS) were identical with European samples from the Czech Republic and Danube floodplain forests (data not shown). This provides the first indication of the likely origin of the Czech focus of fascioloidosis. However, detailed study of genetic interrelationships of global *F. magna* populations should be assessed by multilocus population genetic markers, such as microsatellites, polymorphic and codominant markers which were recently designed specifically for giant liver fluke [43] and can provide more detailed population structuring.
